# MicroRNA‐142 regulates osteoblast differentiation and apoptosis of mouse pre‐osteoblast cells by targeting bone morphogenetic protein 2

**DOI:** 10.1002/2211-5463.12929

**Published:** 2020-07-31

**Authors:** Bing Luo, Jiafu Yang, Yi Yuan, Pandeng Hao, Xiaoyan Cheng

**Affiliations:** ^1^ Department of Orthopedics The Affiliated Traditional Chinese Medicine Hospital of Southwest Medical University Luzhou China; ^2^ Department of Anesthesiology Weifang People's Hospital Weifang China

**Keywords:** apoptosis, BMP2, differentiation, miR‐142, proliferation

## Abstract

Osteoporosis is a common disease that can seriously impair the physical and mental health of the elderly. However, current treatment is unsatisfactory due to the lack of effective therapeutic targets. Abnormal expression and involvement of microRNA‐142 (miR‐142) have been identified in many diseases, including bone‐related diseases. Herein, we explored the effect of miR‐142 on the viability, differentiation and apoptosis of the mouse preosteoblast cell line MC3T3‐E1. We observed that the viability of MC3T3‐E1 cells was significantly inhibited or promoted after transfection of miR‐142 mimic or inhibitor, respectively. The apoptotic rate was dramatically increased by miR‐142 mimic and decreased by inhibitor compared with the negative control group. Bcl‐2 expression was down‐regulated in the miR‐142 mimic group and up‐regulated in the miR‐142 inhibitor group, whereas levels of cleaved caspase‐3 and Bax were increased in the miR‐142 mimic group and reduced in the miR‐142 inhibitor group. Expression changes of Runx2 and Osteocalcin suggest that miR‐142 inhibits the differentiation of osteoblast cells. Moreover, the luciferase reporter assay was used to verify that bone morphogenetic protein 2 (BMP2) is a target of miR‐142. Overexpression of BMP2 repressed the proapoptotic effect of miR‐142 mimic, whereas knockdown of BMP2 abolished the inhibitory effect of miR‐142 inhibitor on the apoptosis of MC3T3‐E1 cells. Furthermore, up‐regulation or down‐regulation of miR‐142 dramatically decreased or increased the ratio of p‐Smad1/5/Smad1 and p‐Smad1/5/Smad5, respectively. Collectively, our results imply that miR‐142 might influence the viability and differentiation of osteoblast cells by regulating BMP2 and BMP/Smad signaling.

## Graphical abstract

Here, we explored the effect of microRNA‐142 (miR‐142) on the viability of a mouse preosteoblast cell line. Cell viability was inhibited or promoted after transfection of miR‐142 mimic or inhibitor, respectively. Moreover, bone morphogenetic protein 2 (BMP2) is a target of miR‐142. Our results imply that miR‐142 might influence viability and differentiation of osteoblast cells by regulating BMP2 and BMP/Smad signaling.

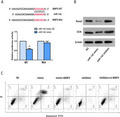

**Abbreviations**
*A*: absorbance
ALP: alkaline phosphatase
BMP2: bone morphogenetic protein 2
BMP2‐Mut: BMP2 3′ UTR sequence with mutations in miRNA‐142 binding site
BMP2‐WT: BMP2 3′ UTR wild‐type sequence
miR‐142: microRNA‐142
miRNA: microRNA
NC: negative control
OCN: osteocalcin
qPCR: quantitative PCR
Runx2: Runt‐related transcription factor‐2
SD: standard deviation
TGF‐β: transforming growth factor‐β

Osteoporosis is a bone disease featured by declined bone mass, destruction of bone structure, increased bone fragility and easy fracture [1, 2, 3, 4]. It often occurs because of the destroyed bone homeostasis: reduced bone formation due to the declined osteoblast and functional defects and exceeding bone resorption induced by increased osteoclast, as well as enhanced activation [5]. Currently, the therapy for osteoporosis mainly focuses on restoring and maintaining the balance between bone formation and bone resorption [6]. However, the current treatment is still unsatisfactory due to the lack of effective therapeutic targets. Hence clarifying the molecular pathogenesis of osteoporosis and identifying effective therapeutic targets are of great significance for improving osteoporosis treatment.

MicroRNAs (miRNAs) are a series of short noncoding RNAs (21–25 nucleotides) that regulate the translation of mRNAs [7, 8]. Recently, many dysregulated miRNAs that are associated with the progression of osteoporosis have been recognized [9]. For example, microRNA‐422a (miR‐422a) and miR‐133a have been recognized as potential biomarkers in circulating monocytes for postmenopausal osteoporosis [10]. miR‐223 promoted the differentiation of mouse osteoclast by down‐regulating NF1‐A (nuclear factor 1‐A) expression [11]. miR‐155 could improve osteoclast activity possibly by targeting Src homology 2‐containing inositol phosphatase [12]. Kim *et al*. [13] found that miR‐182 was up‐regulated in patients with osteoporosis and could suppress the expression of FoxO1 and induce the damage of the protective system of oxidative stress. miR‐142 has been illustrated to be involved in the malignancy of breast cancer stem cells by regulating the WNT signaling pathway [7]. Wang *et al*. [14] have illustrated that miR‐142 plays an inhibitory part in airway smooth muscle cell growth and a promoting role in the apoptosis airway during remodeling in asthmatic rats by suppressing the transforming growth factor‐β (TGF‐β)‐dependent EGFR signaling pathway. In osteosarcoma, miR‐142 inhibits the growth and causes apoptosis of osteosarcoma cells via regulating retinoblastoma‐associated protein [8]. More importantly, Lou *et al*. [15] have illustrated that miR‐142 facilitates the osteoclastogenesis of bone marrow‐derived macrophages via targeting PTEN and regulating the phosphatidylinositol‐3‐kinase (PI3K)/Akt/FoxO1 pathway, suggesting the involvement of miR‐142 in osteoporosis. However, the effect of miR‐142 on the differentiation and apoptosis of preosteoblast cells and the corresponding mechanism remains unclear because of the complex molecular mechanism of osteoporosis.

Herein, we investigated the potential function of miR‐142 on the viability, differentiation and apoptosis of preosteoblast MC3T3‐E1 cells. Furthermore, we studied whether bone morphogenetic protein 2 (*BMP2*) is a target of miR‐142. Our findings demonstrated that miR‐142 represses cell growth and differentiation and promotes apoptosis of MC3T3‐E1 cells partially via targeting *BMP2*.

## Materials and Methods

### Cell line

The mouse preosteoblast cell line MC3T3‐E1 Subclone 14 was purchased from American Type Culture Collection (Manassas, VA, USA) and maintained in alpha minimum essential medium, which was added to 10% FBS, 100 U·mL^−1^ penicillin, and 100 g·mL^−1^ streptomycin. β‐Glycerophosphate (10 mm·L^−1^) and ascorbic acid (50 μg·mL^−1^; Sigma) were used to induce MC3T3‐E1 cell differentiation.

### Transfection

The cells were transfected with transfection reagent [negative control (NC) group], miR‐142 mimic NC sequence (scrambled sequence; miR‐142 mimic NC group), miR‐142 mimic (miR‐142 mimic group), miR‐142 inhibitor NC sequence (scrambled sequence; miR‐142 inhibitor NC group), miR‐142 inhibitor (miR‐142 inhibitor group), miR‐142 mimic+pcDNA3.1‐*BMP2* (miR‐142 mimic+*BMP2* group) or miR‐142 inhibitor+si‐*BMP2* (miR‐142 inhibitor+si‐BMP2 group), which were provided by GenePharma Co. (Shanghai, China), using Lipofectamine 2000 accordingly. After 24‐h transfection, the following experiments were conducted.

### MTT assay

After 24‐h transfection, MC3T3‐E1 cells (10^5^ cells·mL^−1^) were seeded into a 96‐well plate. After the cells were attached, the medium was exchanged with fresh medium followed by being added with 10 mmol·L^−1^ β‐glycerophosphate and 50 μg·mL^−1^ ascorbic acid. Afterward, cell transfection was conducted as described earlier. Forty‐eight hours later, 20 μL MTT solution (Sigma Aldrich, St Louis, MO, USA) was added to each well. The supernatant was discarded after incubation for 4 h in an incubator with 5% CO_2_. Hereafter, the plate was added with DMSO (200 μL per well) and incubated for 10 min on a shaker. When blue‐violet particles crystallized and fully dissolved, the *A*_490_ was assessed using a microplate reader.

### Flow cytometry

After 24‐h transfection, cells were seeded into six‐well plates and cultured for 24 h. Then cells were harvested and treated by 0.25% trypsin and then washed by precooled PBS. Then the cells were suspended using the binding buffer, and the cell concentration was adjusted to about 1–5 × 10^6^ cells·mL^−1^. Cell suspension (100 µL) and Annexin V/FITC (5 µL) were added into a tube and maintained for 5 min, keeping away from light. Hereafter, propidium iodide (PI) stain and PBS were added into the tube. Finally, a flow cytometer (BD Biosciences, San Jose, CA, USA) was applied to detect the results, and flowjo 10 software (Tree Star, OR, USA) was used to analyze the results.

### Dual‐luciferase reporter gene assay

The TargetScan website (http://www.targetscan.org) was used to forecast the targets of miR‐142 and the potential target sites. A *BMP2* 3′ UTR wild‐type sequence (*BMP2*‐WT) and a *BMP2* 3′ UTR sequence with mutations in the miRNA‐142 binding site (BMP2‐Mut) were synthesized. Then BMP2‐WT and BMP2‐Mut were ligated into the pmirGLO vector (Promega, USA) to generate pmirGLO‐*BMP2*‐WT and pmirGLO‐BMP2‐Mut vectors. Then we transfected pmirGLO‐WT+miRNA‐142 mimic NC, pmirGLO‐WT+miRNA‐142 mimic, pmirGLO‐Mut+miRNA‐142 mimic NC or pmirGLO‐Mut+miRNA‐142 mimic, respectively. After 48‐h transfection, total proteins were extracted, and Dual‐Luciferase Reporter Assay Kit (Promega Corporation, Madison, WI, USA) was used to perform the luciferase activity assays.

### Quantitative RT‐PCR analysis

#### miRNA extraction and quantitative RT‐PCR analysis

Total RNA was isolated from the MC3T3‐E1 cells using TRIzol (Invitrogen, Carlsbad, CA, USA). Then the obtained RNA was reverse transcribed using a Mir‐X miRNA First Strand Synthesis Kit with special stem‐loop primer for miRNAs (Table 1). Then quantitative PCR (qPCR) was completed using the Mir‐X miRNA qRT‐PCR TB Green Kit (TaKaRa, Dalian, China) and ABI 7500 system (Applied Biosystems, Foster City, CA, USA) to quantify the expression of miR‐142. U6 was used as an internal reference. The data were calculated using a $2^{‐\Delta \Delta C_{t}}$ method.

**Table 1:** The primers used for quantitative real‐time RT‐PCR.

Primer name	Sequences
RT primers	
miR‐142 RT primer	5′‐GTCGTATCCAGTGCGTGTCGTGGAGTCGGCAATTGCACTGGATACGACAGGTAGC‐3′
U6 RT primer	5′‐CGCTTCACGAATTTGCGTGTCAT‐3′
PCR primers	
miR‐142 forward	5′‐CATAAAGTAGAAAGCA‐3′
miR‐142 reverse	5′‐CAGTGCGTGTCGTGGAGT‐3′
U6 forward	5′‐GCTTCGGCAGCACATATACTAAAAT‐3′
U6 reverse	5′‐CGCTTCACGAATTTGCGTGTCAT‐3′
BMP2 forward	5′‐GAGAAGCTAGAGTCGCGGAC‐3′
BMP2 reverse	5′‐GTCCGCGACTCTAGCTTCTC‐3′
OCN forward	5′‐TCTGACAAAGCCTTCATGTCC‐3′
OCN reverse	5′‐AAATAGTGATACCGTAGATGCG‐3′
Runx2 forward	5′‐TGGTAAAGGCTCAGGCATGG‐3′
Runx2 reverse	5′‐AACAGAGAGCGAGGGGGTAT‐3′

#### Detection of *BMP2*, osteocalcin and Runt‐related transcription factor‐2 expression

The cDNA was formed using PrimeScript RT reagent Kit (TaKaRa, Dalian, China) with gDNA Eraser with the isolated total RNA as template. The mRNA levels of *BMP2*, osteocalcin (*OCN*) and Runt‐related transcription factor‐2 (*Runx2*) were examined by qPCR using SYBR Premix Ex Taq II kit (TaKaRa) on an ABI 7500 system according to the manufacturer's description. GAPDH was used as the reference.

### Alkaline phosphatase activity test

MC3T3‐E1 cells were inoculated into a six‐well plate. After the cells were attached, the medium was replaced and added with 10 mm β‐glycerophosphate and 50 μg·mL^−1^ ascorbic acid. After being cultivated for 10 days, the cells were lysed and the supernatant was harvested. The concentration of the protein in the supernatant was measured using a BCA kit. Alkaline phosphatase (ALP) content was measured using an Alkaline Phosphatase Assay Kit (Beyotime Biotechnology, Shanghai, China) according to the description. The *A* value was measured by a microplate reader at 405 nm, and the relative ALP activity was computed on the basis of the measured *A* value.

### Western blot

Radioimmunoprecipitation assay lysate plus protease inhibitor was used to extract the protein in cells. Then the proteins were separated by SDS/PAGE followed by being transferred to the poly(vinylidene difluoride) membranes (Millipore, Bedford, MA, USA). Afterward, the membranes were probed with primary antibodies and secondary antibody successively. The primary antibodies used were Bcl‐2 (1 : 1000, 3498; CST, Beverly, MA, USA), cleaved caspase‐3 (1 : 1000, 9661; CST), Bax (1 : 1000, 2772; CST), RUNX‐2 (1 : 1000, 8486; CST), OCN (1 : 1000, 23418‐1‐AP; PTG), BMP2 (1 : 1000, 18933‐1‐AP; PTG), p‐Smad1/5 (1 : 1000, 9516; CST), Smad1 (1 : 1000, 9743; CST), Smad5 (1 : 1000, 12534; CST) and β‐Actin (1 : 1000, 4970; CST). The proteins were detected applying an enhanced chemiluminescence detection kit (Pierce Biotechnology, Inc., Rockford, IL, USA). The density of the bands was quantified using the quantity one software (Bio‑Rad, Hercules, CA, USA) with β‐Actin as the reference.

### Statistical analysis

All of the values were shown as mean ± standard deviation (SD). One‐way ANOVA with a Dunnett's or Bonferroni's *post hoc* test was performed for comparisons among three or more groups. Two‐way ANOVA followed by *post hoc* Bonferroni's test was conducted to analyze the data obtained in dual‐luciferase reporter gene assay. The result was statistically significant when *P* < 0.05.

## Results

### Up‐regulation of miR‐142 inhibits MC3T3‐E1 cell viability

**Fig. 1:**
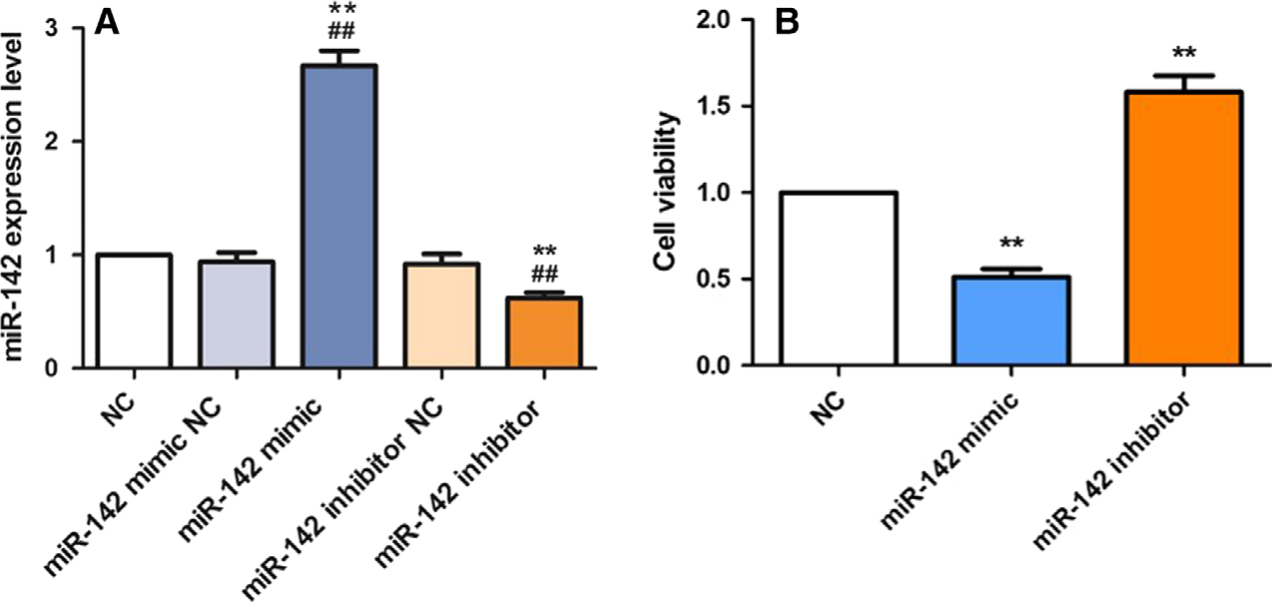
miR‐142 affects MC3T3‐E1 cell viability. (A) The mRNA expression of miR‐142 in MC3T3‐E1 cells after treatment with miR‐142 mimic or inhibitor was detected by qRT‐PCR. One‐way ANOVA with a Bonferroni's *post hoc* test was used for statistical analysis. **P < 0.01 versus NC; ^##^ *P* < 0.01 versus miR‐142 mimic NC or miR‐142 inhibitor NC, accordingly. (B) Cell viability was repressed by miR‐142 mimic and raised by miR‐142 inhibitor. One‐way ANOVA with a Dunnett's *post hoc* test was used for statistical analysis. Error bars represent SD. *n*= 6. **P < 0.01 versus NC.

To explore the influence of miR‐142 on MC3T3‐E1 cells, we up‐regulated or down‐regulated its expression levels by miR‐142 mimic and miR‐142 inhibitor, respectively. As exhibited in Fig. 1A, the mRNA expression level of miR‐142 in MC3T3‐E1 cells was dramatically enhanced in the miR‐142 mimics group (*P* < 0.01) and decreased in the miR‐142 inhibitor group (*P* < 0.01). We observed that there is no significant difference of miR‐142 expression among NC, miR‐142 mimic NC and miR‐142 inhibitor NC groups. Therefore, in the following experiments, only the NC group was included, whereas the miR‐142 mimic NC and miR‐142 inhibitor NC groups were not included. Via MTT assay, we found that MC3T3‐E1 cell viability was decreased after up‐regulation of miR‐142 by miR‐142 mimic (*P* < 0.01; Fig. 1B). In contrast, MC3T3‐E1 cell viability was increased after transfection of miR‐142 inhibitor (*P* < 0.01; Fig. 1B). These observations implied that miR‐142 might act as an inhibitor in MC3T3‐E1 cell viability during osteoporosis progression.

### Up‐regulation of miR‐142 promotes apoptosis of MC3T3‐E1 cells

**Fig. 2:**
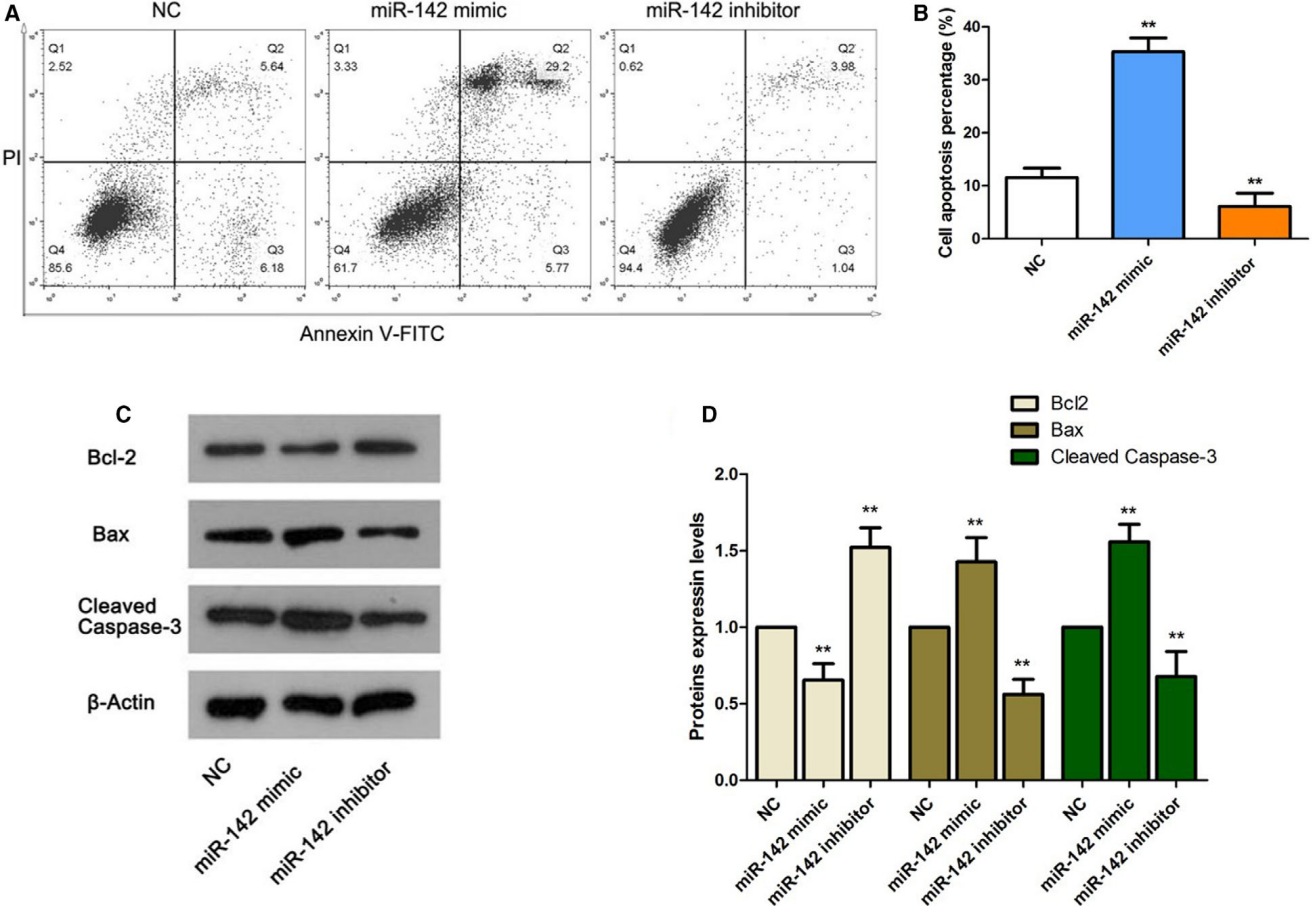
Proapoptotic effect of miR‐142 on MC3T3‐E1 cells. (A) Representative dot plot diagrams of flow cytometry. (B) The apoptosis rate of MC3T3‐E1 cells. (C) The protein expressions of apoptotic‐related proteins. (D) The relative expression levels of apoptotic‐related proteins in (C). One‐way ANOVA with a Dunnett's *post hoc* test was used for statistical analysis. Error bars represent SD. *n* = 6. **P < 0.01 versus NC group.

The apoptosis rate of MC3T3‐E1 cells was dramatically higher in the miR‐142 mimic group (34.97% ± 0.54%) than that in the NC group (11.82% ± 0.83%). However, an obvious decrease was observed in the miR‐142 inhibitor group (5.02% ± 0.96%) compared with the NC group (11.82% ± 0.83%) (*P* < 0.01; Fig. 2A,B). Afterward, the protein expression changes of apoptotic‐related proteins were determined by western blot. The results revealed that the level of Bcl‐2 was reduced, whereas the levels of Bax and active‐caspase‐3 were notably increased in the miR‐142 mimic group compared with the NC group (*P* < 0.01; Fig. 2C,D). In the miR‐142 inhibitor group, the enhanced Bcl‐2 level and declined Bax and active‐caspase‐3 levels were observed (*P* < 0.01; Fig. 2C,D). These findings demonstrated that up‐regulation of miR‐142 facilitated MC3T3‐E1 cell apoptosis and changed the expression of apoptosis‐related proteins.

### Up‐regulation of miR‐142 suppressed ALP activity and decreased the expression of osteoblast differentiation factors Runx2 and OCN

**Fig. 3:**
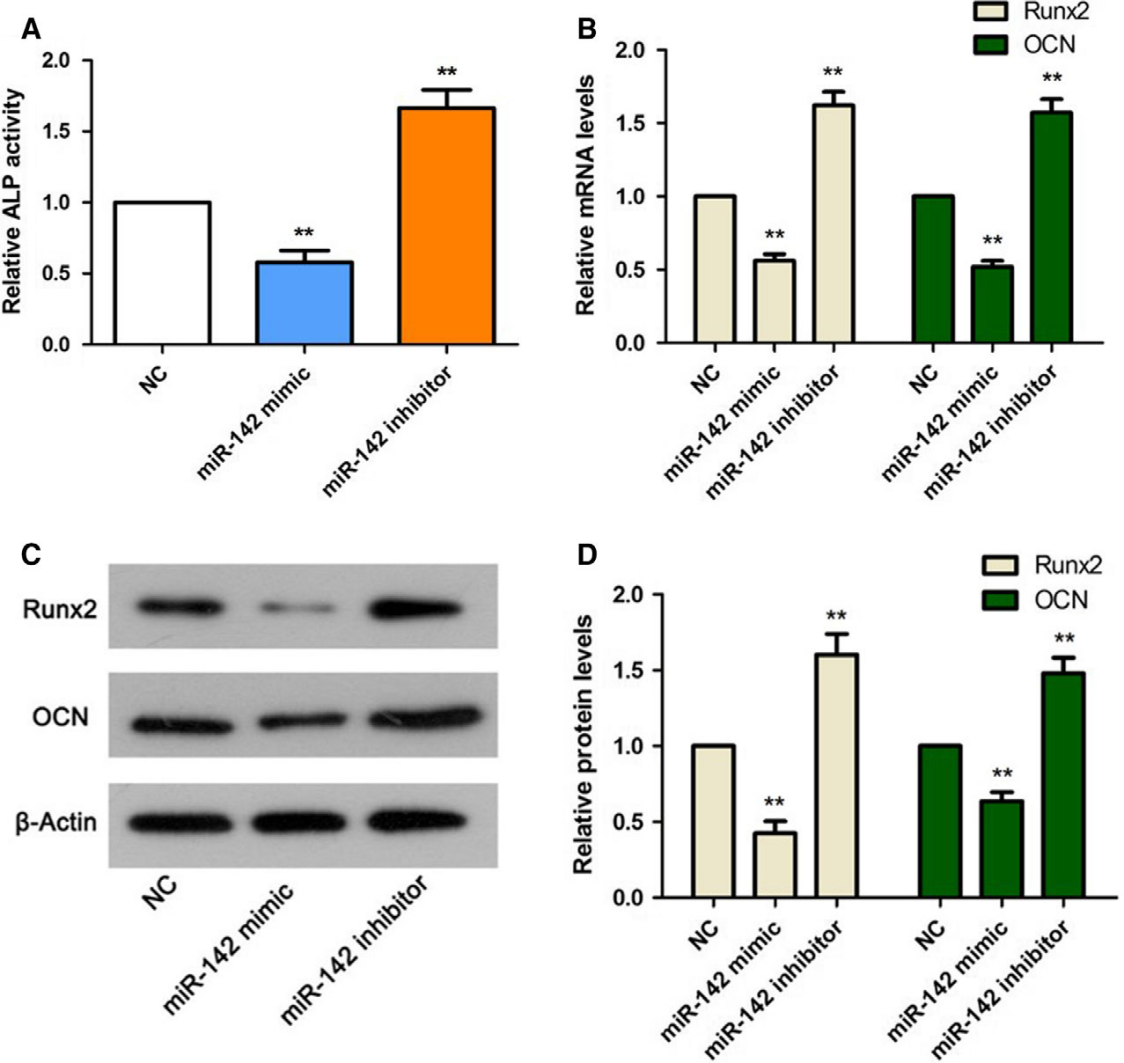
miR‐142 reduced ALP activity and inhibited MC3T3‐E1 cell osteoblast differentiation. (A) ALP activity is reduced in the miR‐142 mimic group and increased in the miR‐142 inhibitor group. (B) The mRNA expression levels of *Runx2* and *OCN* are detected by qRT‐PCR. (C) The protein expression of Runx2 and OCN was determined by western blot. (D) The relative expression levels of Runx2 and OCN in (C) were quantified using Quantity One software. One‐way ANOVA with a Dunnett's *post hoc* test was used for statistical analysis. Error bars represent SD. *n*= 6. **P < 0.01 versus NC group.

After transfection, the ALP activity was dramatically decreased or increased in the miR‐142 mimic and miR‐142 inhibitor groups, respectively (*P* < 0.01; Fig. 3A). Then the levels of osteoblast differentiation factors were determined by qPCR and western blot. We identified that Runx2 and OCN were declined both at mRNA and protein levels after up‐regulating the level of miR‐142 by miR‐142 mimic (*P* < 0.01; Fig. 3B–D). In contrast, decreasing the expression of miR‐142 by the inhibitors dramatically enhanced Runx2 and OCN expression in MC3T3‐E1 cells (*P* < 0.01; Fig. 3B–D), indicating that miR‐142 exhibited a repressive effect on osteoblast differentiation.

### *BMP2* is a target of miR‐142

**Fig. 4:**
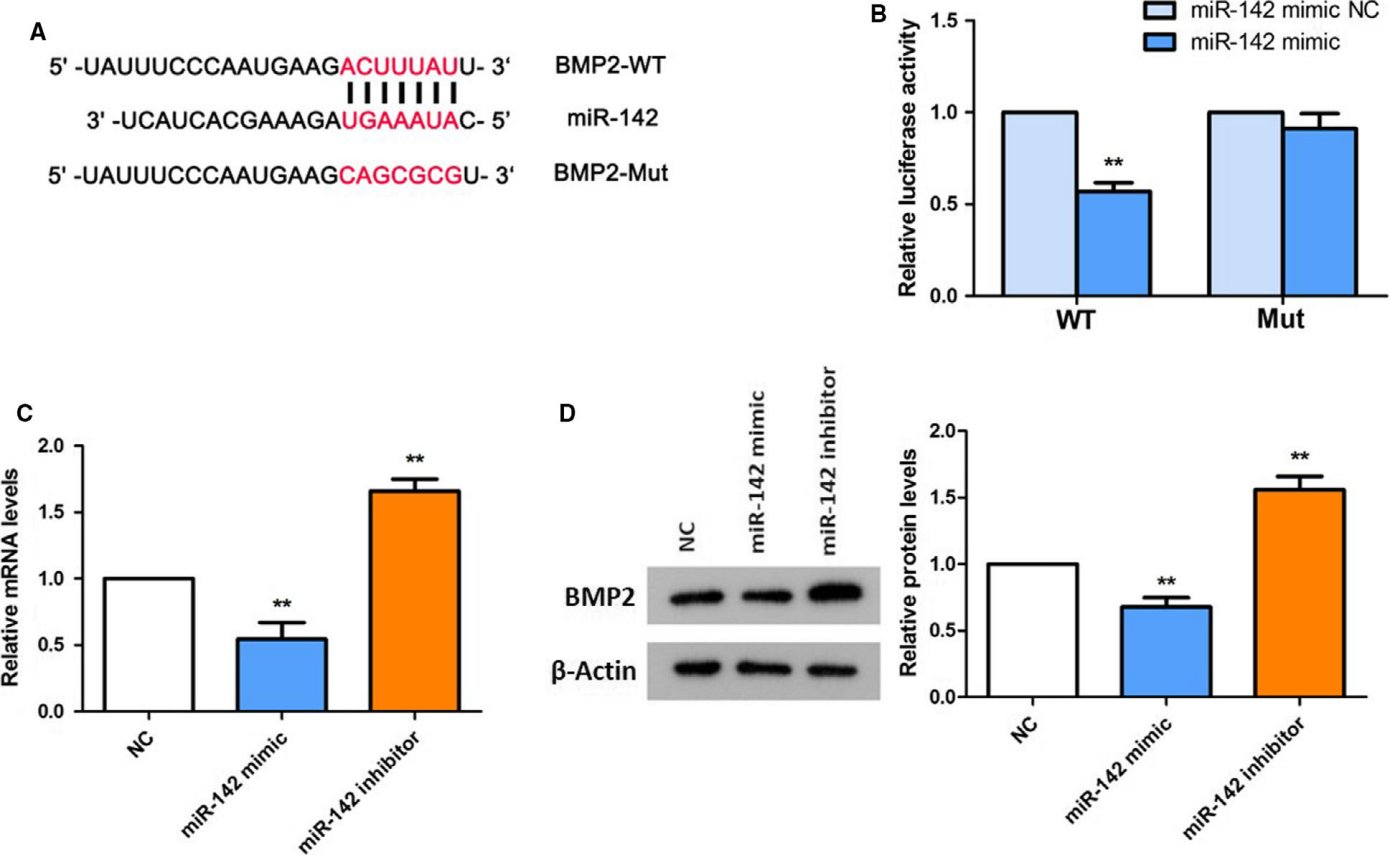
miR‐142 suppresses BMP2 expression by targeting its 3′ UTR. (A) The predicted binding sites of miR‐142 within the 3′ UTR of BMP2 and 3′ UTR of BMP2 with mutations in the binding sites. (B) Luciferase activity assay was applied to detect the interaction between miR‐142 and *BMP2*. Two‐way ANOVA followed by *post hoc* Bonferroni's test was used for statistical analysis. *N* = 6. **P < 0.01 versus miR‐142 mimic NC. (C) The mRNA expression of *BMP2* was reduced in the miR‐142 mimic group and enhanced in the miR‐142 inhibitor group. (D) The protein expression level of BMP2 was decreased in the miR‐142 mimic group and increased in the miR‐142 inhibitor group. One‐way ANOVA with a Dunnett's *post hoc* test was used for statistical analysis. Error bars represent SD. *n*= 6. **P < 0.01 versus NC.

For the purpose of further exploring the possible mechanism underlying the involvement of miR‐142 in cell viability and differentiation, the targets of miR‐142 were forecasted by TargetScan algorithm. We found that *BMP2* was a potential target of miR‐142. The forecasted binding sites between miR‐142 and BMP2 were shown in Fig. 4A. Then a luciferase assay was conducted and showed that miR‐142 mimic exhibited almost no effect on pmirGLO‐Mut, whereas miR‐142 mimic notably decreased the luciferase activity of pmirGLO‐WT in MC3T3‐E1 cells (*P* < 0.01; Fig. 4B). Moreover, we found that the expression level of BMP2 was decreased in the miR‐142 mimic group and increased in the miR‐142 inhibitor group (*P* < 0.01; Fig. 4C,D). Our results verified that miR‐142 could bind to *BMP2* and suggested that miR‐142 possibly affected the viability, apoptosis and differentiation of MC3T3‐E1 cells by negatively regulating BMP2.

### The effect of the miR‐142/BMP2 axis on the apoptosis of MC3T3‐E1 cells

**Fig. 5:**
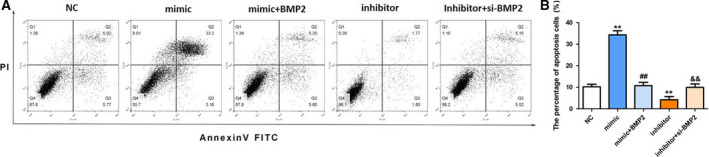
The effect of miR‐142 and BMP2 on the apoptosis of MC3T3‐E1 cells. (A) Representative dot plot diagrams of flow cytometry. (B) The apoptosis rate of MC3T3‐E1 cells. One‐way ANOVA with a Bonferroni's *post hoc* test was used for statistical analysis. Error bars represent SD. *n*= 3. **P < 0.01 versus NC; ^##^ *P* < 0.01 versus miR‐142 mimic; ^&&^ *P* < 0.01 versus miR‐142 inhibitor.

To determine whether BMP2 mediated the effect of miR‐142 on the apoptosis of MC3T3‐E1 cells, we altered the expression of BMP2 by pcDNA3.1‐BMP2 or si‐BMP2. The results revealed that overexpression of BMP2 repressed the proapoptotic effect of miR‐142 mimic on MC3T3‐E1 cells (*P* < 0.01; Fig. 5A,B). In contrast, knockdown of BMP2 abolished the inhibitory effect of miR‐142 inhibitor on the apoptosis of MC3T3‐E1 cells (*P* < 0.01; Fig. 5A,B). These outcomes indicated that miR‐142 exerts its effect on the apoptosis of MC3T3‐E1 cells partially by targeting BMP2.

### Up‐regulation of miR‐142 repressed the BMP/Smad signaling pathway

**Fig. 6:**
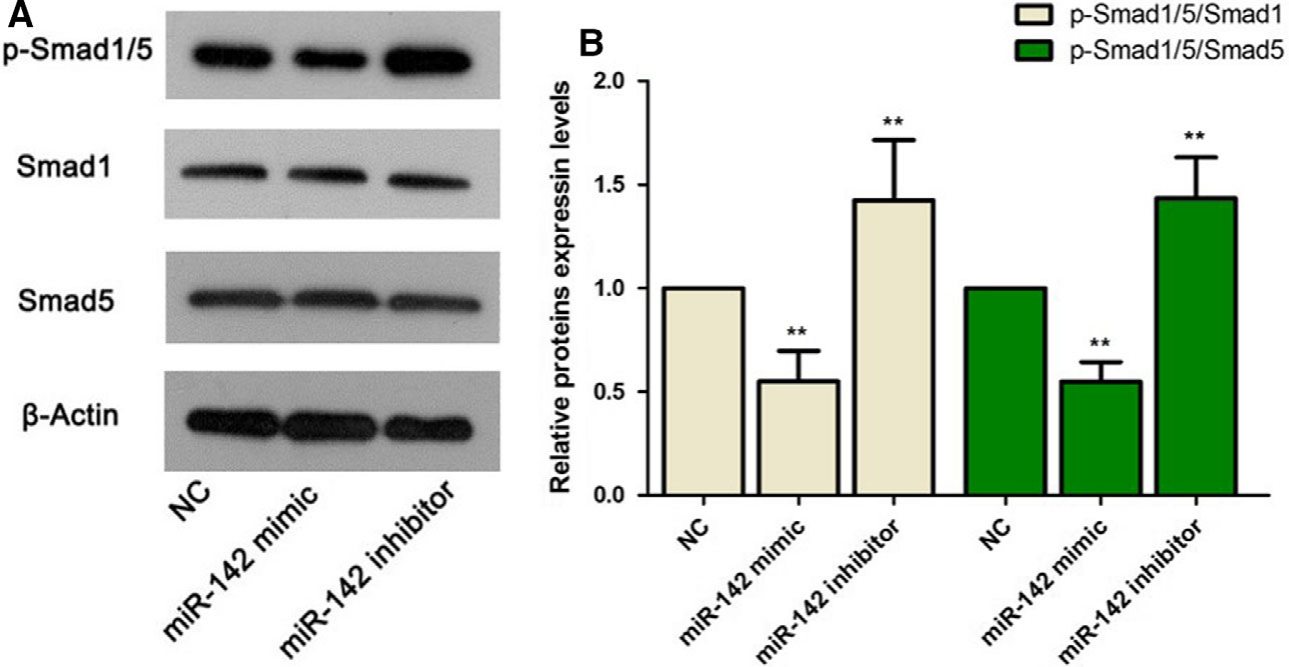
The protein expression levels of p‐Smad 1/5, Smad1 and Smad5. (A) Western blot analysis was used to detect the protein expression levels of p‐Smad 1/5, Smad1 and Smad5. (B) The relative expression levels of p‐Smad 1/5/Smad1 and p‐Smad 1/5/Smad5 were calculated. One‐way ANOVA with a Dunnett's *post hoc* test was used for statistical analysis. Error bars represent SD. n = 6. **P < 0.01 versus NC.

By western blot, we found that the expression levels of Smad1 and Smad5 were almost not changed despite the up‐regulation or down‐regulation of miR‐142. However, the ratios of p‐Smad1/5/Smad1 and p‐Smad1/5/Smad5 were noticeably reduced in the miR‐142 mimic group relative to the NC group (*P* < 0.01; Fig. 6A,B). The ratios of p‐Smad1/5/Smad1 and p‐Smad1/5/Smad5 were obviously higher in the miR‐142 inhibitor group than in the NC group (*P* < 0.01; Fig. 6A,B). These results suggested that miR‐142 might be involved in MC3T3‐E1 cell proliferation and differentiation by adjusting the BMP/Smad signaling pathway.

## Discussion

Our data verified that *BMP2* was indeed a target of miR‐142. Enhancing the expression of miR‐142 suppressed cell viability and differentiation, and induced cell apoptosis partially through regulating the BMP/Smad pathway in MC3T3‐E1 cells.

miR‐142 has been recognized as a tumor‐suppressive miRNA in many types of disease, including bone diseases. In pancreatic cancer cells, miR‐142 was found to suppress cell proliferation and invasion partly by targeting hypoxia‐inducible factor 1α (HIF‐1α), an oncogene [16]. In airway smooth muscle cells, miR‐142 played an inhibitory role in cell proliferation and a promoting role in apoptosis, possibly by inhibiting TGF‐β expression [14]. In addition, miR‐142 was also found to regulate the properties of breast cancer stem cells partly by affecting the WNT pathway [7]. Furthermore, it has been reported that miR‐142 repressed the growth and induced the apoptosis of osteosarcoma cells via regulating Rb [8]. In aged bone marrow mesenchymal stem cells, miR‐142 induced the cumulation of reactive oxygen species through repressing pexophagy [17]. Notably, miR‐142 could promote the osteoclastogenesis of bone marrow‐derived macrophages through targeting PTEN and modulating the PI3K/Akt/FoxO1 pathway, suggesting the involvement of miR‐142‐5p in osteoporosis [15]. Here, we identified that enhancement of miR‐142 obviously repressed MC3T3‐E1 cell viability and increased the percentage of cell apoptosis, whereas depletion of miR‐142 exhibited the opposite influence on cell viability and apoptosis. Our results suggest that miR‐142 is an inhibitor of cell proliferation and a promoter in apoptosis in MC3T3‐E1 cells.

ALP is widely distributed in the body and is known as a bone‐specific marker of mineralization [18]. RUNX‐2 was considered as an early biomarker of osteoblastic differentiation, which has been shown to effectuate the expression of osteopontin (OPN) and OCN [19]. OCN is a noncollagenous protein found in bone and dentine, and is known as a bone gamma‐carboxyglutamic acid‐containing protein [20]. Herein, we identified that overexpression of miR‐142 dramatically reduced ALP activity and the protein levels of RUNX‐2 and OCN, whereas depletion of miR‐142 enhanced ALP activity and the levels of RUNX‐2 and OCN. These observations suggested that miR‐142 might take an inhibitory part in osteoblastic differentiation.

BMPs, the central regulators of osteoblast differentiation, are members of the TGF‐β superfamily, which signals via heteromeric type I and type II serine‐threonine kinase receptors and activated Smad1, Smad5 and Smad8 [21]. BMP2, a member of BMPs, has been demonstrated to promote Runx2 transcription [22]. Moreover, Runx2, together with the activated Smads, will stimulate the other genes in differentiating osteoblasts [23]. In human bone mesenchymal stromal cells, *BMP2* was verified to be a target of miRNA‐98 and involved in cell osteogenic differentiation [24]. In our present research, we discovered from the dual‐luciferase reporter gene assay that miR‐142 could directly bind to *BMP2*. Up‐regulation of miR‐142 using miR‐142 mimics dramatically decreased the level of BMP2 in MC3T3‐E1 cells. In contrast, knockdown of miR‐142 obviously enhanced the expression of BMP2. These data demonstrated that BMP2 is a target of miR‐142 and negatively modulated by miR‐142.

The BMP2/Smad signaling pathway takes a vital part in osteoblast differentiation and bone formation [25]. p‐Smad1/5/8/4 has been found to regulate the expression of ALP and Runx2 [25, 26]. Moreover, many researchers have illustrated that inhibition of p‐Smad1/5/8 expression suppressed osteoblast differentiation [25]. Our results showed that overexpression of miR‐142 reduced the expression of p‐Smad1/5, whereas knockdown of miR‐142 enhanced the expression of p‐Smad1/5. These observations demonstrated that miR‐142 might suppress differentiation of preosteoblast MC3T3‐E1 cells partly via exerting a suppressive effect on BMP2/Smad signaling.

In summary, our present results implied that miR‐142 might be involved in the viability and differentiation of preosteoblast cells partly by regulating BMP2 and BMP/Smad signaling. Further *in vivo* assays and the effect of miR‐142 on osteoclasts deserve our further exploration to comprehensively reveal the function of miR‐142 in osteoporosis.

## Conflict of interest

The authors declare no conflict of interest.

## Author contributions

BL and XC designed this study. BL, JY, YY and PH performed the experiments and analyzed the data. BL drafted the manuscript and prepared the figures. XC explained the data and revised the paper. All authors approved the final version of this manuscript.

## Data Availability

The data are available from the corresponding author upon reasonable request.
